# Testing Segmentation Popular Loss and Variations in Three Multiclass Medical Imaging Problems

**DOI:** 10.3390/jimaging7020016

**Published:** 2021-01-27

**Authors:** Pedro Furtado

**Affiliations:** Dei/FCT/CISUC, University of Coimbra, Polo II, 3030-290 Coimbra, Portugal; pnf@dei.uc.pt

**Keywords:** computers in medicine, segmentation, machine learning, deep learning, MRI

## Abstract

Image structures are segmented automatically using deep learning (DL) for analysis and processing. The three most popular base loss functions are cross entropy (crossE), intersect-over-the-union (IoU), and dice. Which should be used, is it useful to consider simple variations, such as modifying formula coefficients? How do characteristics of different image structures influence scores? Taking three different medical image segmentation problems (segmentation of organs in magnetic resonance images (MRI), liver in computer tomography images (CT) and diabetic retinopathy lesions in eye fundus images (EFI)), we quantify loss functions and variations, as well as segmentation scores of different targets. We first describe the limitations of metrics, since loss is a metric, then we describe and test alternatives. Experimentally, we observed that DeeplabV3 outperforms UNet and fully convolutional network (FCN) in all datasets. Dice scored 1 to 6 percentage points (pp) higher than cross entropy over all datasets, IoU improved 0 to 3 pp. Varying formula coefficients improved scores, but the best choices depend on the dataset: compared to crossE, different false positive vs. false negative weights improved MRI by 12 pp, and assigning zero weight to background improved EFI by 6 pp. Multiclass segmentation scored higher than n-uniclass segmentation in MRI by 8 pp. EFI lesions score low compared to more constant structures (e.g., optic disk or even organs), but loss modifications improve those scores significantly 6 to 9 pp. Our conclusions are that dice is best, it is worth assigning 0 weight to class background and to test different weights on false positives and false negatives.

## 1. Introduction

Various medical imaging modalities are used in different settings to form images of the anatomy and physiological processes of some part of the body. After acquisition, segmentation is an image processing functionality useful for advanced computer-aided analysis, measurements and visualizations related to medical procedures. Deep learning has been applied increasingly in that context to automatically learn how to classify and segment the images. Magnetic resonance imaging (MRI) and computer tomography (CT) are most popular for analysis and diagnosis of multiple affections. Examples of deep learning segmentation on those datasets include acute ischemic lesions [1], brain tumors [2], the striatum [3], organs-at-risks in head and neck [4], polycystic kidneys [5], prostate [6] and spine [7]. References [8,9] review applications in more detail. Analysis of eye-fundus images (EFI) to detect lesions is a very different medical imaging context where precise segmentation can help quantify lesions indicative of diabetic retinopathy [10]. In these, and other medical imaging scenarios, segmentation is a very common operation.

Current state-of-the-art segmentation uses deep convolutional neural networks (DCNN). These systems were first developed to classify images, with some popular architectures being VGG [11] and Resnet [12]. The classification DCNN is made of a sequence of encoder convolution stages (convolutions, activations and pooling) that extract and compress features from the image directly into a feature vector. Next, a fully-connected neural network classifies the image based on the feature vector. The segmentation network is a modified DCNN architecture that classifies each pixel (with a segment label) instead of the image. To achieve this the fully connected layers are replaced by a decoder that successively de-convolves until the full image size is restored. The fully convolutional network (FCN) [13] was one of the first well-structured segmentation network architectures. It uses a DCNN as encoder (e.g., VGG) and replaces the final fully-connected layers by up-sampling interpolation layers. U-Net introduced further innovations [14], with de-convolution stages symmetric to the convolution stages (forming a U-shape) instead of interpolation. DeepLab [15] is another highly accurate segmentation network architecture that introduces important innovations. One such innovation is Atrous spatial pyramid pooling (ASPP), which improves segmentation at multiple scales. Another innovation is the use of conditional random fields (CRF) that applies probabilistic graphical models for improved determination of objects boundaries.

**Figure 1:**
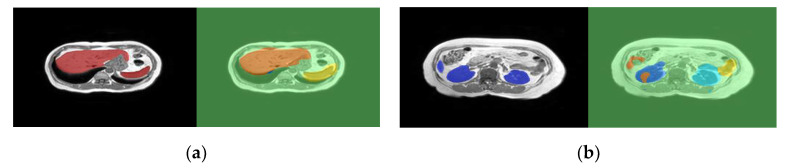
Example magnetic resonance image (MRI) segmentation of independent test images using DeepLabv3 segmentation network. The left of each image is the groundtruth on a black background, the right is the segmentation: (**a**) is a slice showing the liver and spleen; (**b**) is another slice showing the kidneys and a small extremity of the liver.

Learning to segment automatically based on training images and groundtruth segments is a crucial step in segmentation DCNNs. In that process, loss is a fundamental measure of the distance between the current quality of segmentation of training images and the groundtruth segmentations that is used as the basis for backpropagation learning. A loss function that fails to reveal deficiencies in segmentation of specific structures will not learn to segment those structures well. Nevertheless, it is difficult to accurately reflect the loss of different target structures, with different characteristics and occurrences, in a single value (the loss). For that reason, it is common in current state-of-the-art to see a final training validation loss of 1% or less at the same time that incorrections in segmentation of some structures are still quite visible. Figure 1 illustrates this problem with a real case segmentation of two MRI test slices (the groundtruth segments are the left images shown on black background). In this example, the final validation loss was very low (less than 1% cross entropy loss), but imperfections are quite visible, especially in the case of (b).

The most popular loss functions are cross entropy (crossE), dice and sometimes also IoU (a.k.a Jaccard index). However, given different segmentation contexts in medical imaging, the question arises as to which network, loss function and loss function tuning can optimize the results? In this work, we experiment segmentation in the three imaging contexts (MRI, CT and EFI) to understand which of the three most popular loss functions works better, and to evaluate how changing coefficients weights in the formulas might modify the results. We also evaluate whether it would be preferable to always solve one uniclass segmentation for each target structure or the single multiclass segmentation. In order to reach conclusions we run the following experiments: (1) choose best performing network; (2) compare the three loss functions and variations, such as different weights to false positives and negatives and removal of background class from the formula (zero weighting the background); and (3) evaluate also the alternative of simply replacing multiclass by n uniclass segmentation problems. We observed the following: (1) DeeplabV3 was always better than UNet or FCN; (2) dice is the best in average over the three datasets, followed by IoU and finally crossE; (3) variations were useful in different ways in different datasets: while dice was the best in CT of the liver, IoU with specific weight modifications was the best in the MRI dataset and dice without background was the best alternative in the EFI dataset. From those results, we conclude that: (1) dice is the best scoring alternative in average over all datasets; (2) it is useful to consider different weights variations and to tune for a specific context, because we obtained significant scores improvements; (3) the single multiclass problem was preferable to expressing and solving *n* uniclass problems.

We also compare segmentation scores of larger and more constant classes, such as the optic disk or organs. In addition, we compare with scores of small and very changing targets, such as small microaneurysms in eye fundus images. We observe that the optic disk (90%) and organs (77 to 86%) score much higher than the smaller, location and conformation changing lesions (18 to 61%). Modifications to loss functions improved scores of the small lesions by 5 to 15%.

### Related Work

Deep learning revolutionized segmentation. Prior to the use of deep learning (DL), segmentation of organs in MRI and CT would most frequently be based on multi-atlas approaches (e.g., [16] uses 3D models of the liver and probability maps, [17] is based on histograms and active contours to segment the liver, [18] applies watershed and active contours). Since around 2014, deep learning-based segmentation gradually became the norm. In what concerns recent works on segmentation of MR and CT, Zhou [19] achieved top scores using a fully convolutional networks (FCN) by taking 3-D CT images and applying a majority voting scheme on the output of segmentation of 2D slices taken from different image orientations. Reference [20] applied a similar approach to segmentation of the abdomen from MRI sequences, scoring (dice similarity coefficient = DSC) 0.93, 0.73, 0.78, 0.91, 0.56 for spleen, left and right kidney, liver and stomach respectively. Larsson [21] proposed DeepSeg which segments abdominal organs using multi-atlas, Convolutional Neural Networks (CNN) for pixel binary classification and thresholding to keep only largest connected region (JI: 0.9; 0.87; 0.76; 0.84 for liver, spleen, right and left kidney). Reference [22] proposed multi-slice 2D neural network designed in a way that considers information of subsequent slices, plus augmented data and multiview training. Groza [23] presents an ensemble of DL networks with voting, and [24] tests different architectures (U-Net, deeper U-Net with VGG-19, a cascade of two networks). Loss is considered in [25], where the authors proposed improving deep pancreas segmentation in CT and MRI images via recurrent neural contextual learning and “direct” loss function. They propose a Jaccard Loss (JACLoss): “It empirically works better than the cross-entropy loss or the class-balanced cross-entropy loss when segmenting small objects”. Reference [24] also replaced cross-entropy by the dice function to better deal with class imbalance.

Deep learning has also been applied extensively to detection of lesions in eye fundus images. Works include Prentasic et al. [26], Gondal at al. [27], Quellec et al. [28] (exudates, hemorrhages and microaneurisms), Haloi et al. [29], van Grinsven et al. [30], Orlando et al. [31] and Shan et al. [32] (microaneurisms, hemorrhages or both). Some classify small square windows to detect lesions, others extract lesion heat maps from the DCNN and yet others apply segmentation networks directly. In terms of results evaluating segmentation quality, reported sensitivities against one false positive per image (FPI) in some of those works were (HA = hemorrhages, MA = micro-aneurisms, HE = hard exudates, SE = soft exudates): Quellec [28] (HA = 47%; HE = 57%; SE = 70% and MA = 38%), Gondal [27] (HA = 50%; HE = 40%; SE = 64% and MA = 7%) and Orlando [31] (HA:50%, MA: 30%).

The loss function is based on a metric, and the problem of metrics in general is mentioned in [33]: “many scores are artificially high simply because the background is huge and hence the term TN (true negatives) is also huge”. In what concerns study of the loss function, the work in [34] compares alternative loss functions for the binary problem only (one class). In a different context, [35] investigated a modified loss function that is useful in our work as well. Ref. [36] investigates the use of prior information, which is the use of information regarding acceptable shapes, conformations, textures or colors to enhance loss function.

Comparing to all the related works we just reviewed, and considering the three different contexts that we have chosen (MRI and CT of organs, and EFI lesions) we ask the question of whether any of the three most popular loss functions has best results along the three contexts, whether different weighting of formula coefficients might be worth and how they compare.

## 2. Materials and Methods

In this section, we first introduce the MRI, CT and EFI data used in our experimental work, then we describe our investigative methods. We analyze metrics and their limitations, describing the loss function variations and alternatives based on that analysis. Then we describe an experimental setup to evaluate the quality of segmentation with the loss alternatives.

### 2.1. The Datasets

**Figure 2:**
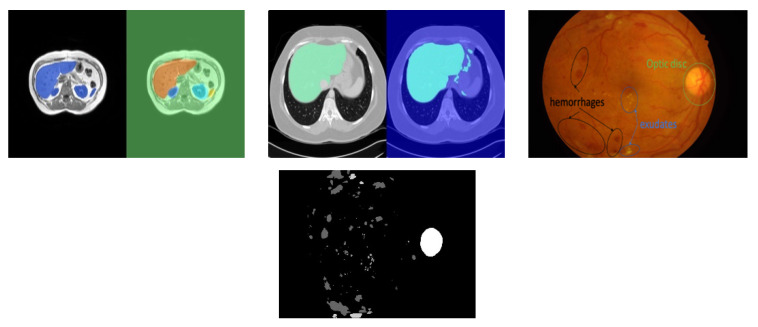
Illustrative examples from the three datasets used. Left: MRI segmentation of liver, spleen, left and right kidneys (groundtruth + segmented); Center: computer tomography (CT) segmentation of liver (groundtruth + segmented); Right: eye fundus image (EFI) with indication of some lesions and example groundtruth.

The three datasets used in this experiment are illustrated briefly in Figure 2. The magnetic resonance imaging (MRI) data used in our experiments are a set of scans available in [37]. The dataset in [37] includes 120 DICOM scans (40 T1-DUAL in phase, 40 T1-DUAL out phase and 40 T2-SPIR), obtained from healthy patients (routine scans, no tumors, lesions or any other diseases). These scans capture abdominal organs (liver, the two kidneys and spleen). In this work, we report our results for 40 in-phase sequences of the T1-DUAL fat suppression protocol. The sequences were acquired by a 1.5T Philips MRI, which produces 12-bit DICOM images with a resolution of 256 × 256. The inter-slice distance ISDs varies between 5.5–9 mm (average 7.84 mm), x-y spacing is between 1.36–1.89 mm (average 1.61 mm) and the number of slices per scan is between 26 and 50 (average 36). Train, test and validation data independent from each other were always obtained by dividing the patients into those subsets. To ensure independent testing, in each run the patients sequences (dataset) were divided into training and testing sequences using a ratio 80%/20%. To obtain multiple runs, the patient sequences were divided randomly into five folds such that each fold has 20% of all patients. In each run, one of the folds was assigned to testing and the remaining folds were used for training. Data augmentation was also added after we verified that it contributes to improved scores, by increasing diversity and size of the dataset. Data augmentation was defined based on random translations of up to 10 pixels, random rotations up to 10 degrees, shearing up to 10 pixels and scaling up to 10%.

We also use a computer tomography (CT) dataset composed of upper abdomen sequences from 40 different patients [37]. The images were acquired using equipment—Philips SecuraCT (Phillips, Amsterdam, Netherlands), 16 detectors, Philips Mx8000, 64 detectors, Toshiba AquilionOne, 320 detectors (equipped with spiral CT). Subjects were all healthy (livers did not exhibit lesions or disease). A contrast agent was used, the abdomen sequences obtained at hepatic phase, i.e., 70–80 s p.i. or 50–60 s after bolus tracking. In this phase, the liver parenchyma enhances through blood supply by the portal vein, resulting in some potential enhancement of the hepatic veins. The resulting 2874 slices have a resolution of 512 × 512, XY spacing 0.7 to 0.8 mm and inter slice distance 3 to 3.2 mm.

The EFI dataset we used is IDRID [10], a dataset that is publicly available for the study of automated detection of diabetic retinopathy and segmentation of characteristic lesions (microaneurysms, hemorrhages, hard exudates, soft exudates), plus the optic disc. It has groundtruth labelled data for each of 83 eye fundus images (EFI), where most images have a large number of instances of each specific lesion, and the groundtruths represent the class that should be assigned to each individual pixel. IDRID contains the pixel groundtruths for micro-aneurisms, hemorrhages, exudates (hard and soft) and the optic disk. The equipment used to acquire the images was a Kowa VX-10 alpha digital fundus camera with 50-degree field of view (FOV), centered near the macula. Image resolution was 4288 × 2848, saved as jpg. Experts validated the quality of the images and their clinical relevance.

### 2.2. Method

#### 2.2.1. Discussing Metrics and Loss

Both segmentation evaluation metrics and loss function are expected to quantify the difference (error) between the groundtruth (GND), representing a correct segmentation of the image, and the segmentation output (SEG). The loss f(SEG, GND) is a single quantity between 0 and 1, and the quality of segmentation is (quality = 1 − loss). SEG and GND are labelmaps, i.e., each position (pixel) in the labelmap is a class label. In most bibliography, metrics are defined considering a binary classification problem that classifies into two classes: positive (P), with the meaning “is”, and negative (N), with the meaning “is not”. The quantities TP, TN, FP and FN correspond to the number of pixels that are true positives, true negatives, false positives and false negatives, respectively. Given those quantities, some of the most frequently used metrics are:

Accuracy (ac) = (TP + TN)//TP + TN + FP + FN);(1)

Sensitivity (se) = recall = True Positive Rate (TPR) = TP/(TP + FN)(2)

Specificity (sp) = TN/(TN + FP)(3)

Precision (p) = TP/(TP + FP)(4)

False Positive Rate (FPR) = FP/(FP + TN)(5)

ROC, a plot of TPR vs. FPR, and AUC, the area under the curve of ROC(6)

IoU = JI = TP/(TP + FN + FP)(7)

Dice (dice) = DSC = 2TP/(2TP + FP + FN) = 2JI/(JI + 1), which is highly correlated with JI(8)

In multiclass problems, we can apply the same formulas, but considering the following quantities instead: a TP pixel is a pixel that belongs to one class c different from background in groundtruth and also in the segmentation; a TN pixel is a pixel that belongs to background in both groundtruth and segmentation; an FP pixel is a pixel that belongs to background in groundtruth but is classified as some other class c in segmentation; an FN pixel is a pixel that belongs to some class c different from background in groundtruth but is then classified as background.

The following three observations are important reasons why the metrics defined in Equations (1)–(8) can fail to evaluate segmentation correctly in many medical imaging contexts:

(1) The number TP is always huge in all metrics, because TP of background pixels is huge. As a consequence, all metrics (1) to (8) report high scores regardless of the actual quality of segmentation of individual classes if evaluated over all pixels;
(2) TN is also huge because it includes a huge number of background pixels that are well classified. It means that specificity (SP), FPR, ROC and AUC do not evaluate the quality of segmentation of individual classes well;
(3) Sensitivity (a.k.a recall or TPR), although useful because it quantifies the fraction of organ pixels classified correctly as such, fails to capture very important possible deficiencies, because it does not include FP (background classified as organ) in the formula, a frequent occurrence.

The problems identified in (a) and (b) are a consequence not only of class imbalance, but most importantly of the fact that background pixels are much easier to segment (score much higher) than pixels of another target class, because they are more constant across most slices and patients (since they include all pixels “framing” the image except the target class itself). The issue identified in (a) means that it is necessary to use metrics that evaluate each class separately instead of computing them over all pixels, requiring modifications to how Equations (1)–(8) were defined above. Additionally, since (b) and (c) discard many metrics that are inappropriate, the metrics that are left for use arejaccard index (JI), and Dice Sorenson Coefficient (DSC) and precision (which should be used together with recall). Given the observations in (a), these need to be evaluated separately for each class. That means each quantity TP, TN, FP and FN must be replaced by TPc, TNc, FPc and FNc respectively, where c is a class, and the metrics should be obtained and reported separately for each class c.

However, while we can report a different value of JI or DSC for each class when evaluating segmentation quality, the loss function needs to output a single value to be used as delta in backpropagation learning. Therefore, the final loss must be averaged over the loss of each class. This solution is still not perfect because the loss of class “background” is in practice always almost zero (due to (a) and (b)), contributing to push the average loss down, even if specific target classes are not very well segmented. Based on these observations, we define the loss functions and variations to consider in the next sub-section.

#### 2.2.2. Defining Metrics and Variations for Use as Loss Function

Based on the previous analysis, we define a set of loss functions besides cross entropy, and a set of variations and alternatives that may contribute to improve the quality of the learning process. We also include the standard cross entropy as one of the options to compare to.

Cross entropy (*crossE*, the default to compare with): cross-entropy is well-known and the default loss function. Given the set of probabilities p of a single pixel of the segmentation output to be of each possible class, and the real probabilities (one-hot encoding of the class), cross entropy measures dissimilarity between p and q. If *t_i_* and *s_i_* are the groundtruth and the CNN score of each pixel for each class *i* respectively,

(9) $$crossE=-\sum_{i}^{C}t_{i}\log s_{i}$$

By applying a class frequency inverse weight to the value for each pixel, we obtain class-weighted cross-entropy, which is the variant we use and denote as “*crossE*”.

Intersect over the union (*IoU*): *IoU* is a convenient measure of the degree of overlap or match between segmentation-obtained regions and the corresponding groundtruth regions. Given the number of true positives (*TP*), false positives (*FP*) and false negatives (*FN*) in the classification of pixels, loss is (1-*IoU*).

(10) $$IoU\left(loss\right)=1-IoU=1-\frac{TP}{TP+FP+FN}$$

However, since this *IoU* averages over all pixels and we identified the problem with that measurement, *IoU* averaged over the classes is used instead,

(11) $$IoU\left(loss\right)=1-\frac{\sum_{I=1}^{C}IoU_{i}}{C},IoU_{i}=1-\frac{TP_{i}}{TP_{i}+FP_{i}+FN_{i}}$$

Dice (dice): The dice or dice similarity coefficient (*DSC*) is a metric that is highly correlated and can be obtained from *IoU* directly. The loss formula for the dice is:

(12) $$dice\left(loss\right)=1-DSC=1-\frac{2TP}{2TP+FP+FN}$$

As with *IoU* we use an average over classes,

(13) $$dice\left(loss\right)=1-\frac{\sum_{I=1}^{C}dice_{i}}{C},dice_{i}=1-\frac{2TP_{i}}{2TP_{i}+FP_{i}+FN_{i}}$$

Intersect over the union with penalties (*IoUxy*): *IoUxy* is similar to *IoU* but penalizes differently *FP* and *FN* in the denominator of the formula. The resulting formula weighting over classes is:

(14) $$IoU_{xy}\left(loss\right)=1-\frac{\sum_{I=1}^{C}IoU_{xyi}}{C},IoU_{xyi}=1-\frac{TP_{i}}{TP_{i}+\alpha FP_{i}+\beta FN_{i}}$$

In these formulas, *α* and *β* are such that *α* + *β* = 2, *α*, *β* ≥ 0. The question to answer is whether giving different weights to *FN* and *FP* (the two types of unwanted errors) will allow the approach to better segment each organ, and how varying the combination of *α* and *β* affects the result. We evaluate this by means of experimentation.

Loss without considering the background (dice noBK): Since the background is easier to segment than the remaining classes and is also huge, dice noBK is an alternative that removes the background from the loss formula (i.e., it averages loss over all classes except the background). The objective is to try to emphasize the need to segment the other classes well. An experimental approach is necessary to evaluate if this alternative improves the outcome.

Uniclass segmentation: instead of a single multi-class problem with a single segmentation network, we can have one specific segmentation network specializing in segmenting each target class. The potential advantage is that we will be replacing a difficult multi-objective optimization problem [38] (minimize loss of segmentation of each organ) by *n* easier to optimize single objective uniclass problems (each one optimizes segmentation of one organ). Note. however that, on the other hand, in uniclass versions all target classes are marked as background except the one being segmented. An experimental approach is necessary to reach conclusions regarding which alternative scores best, either a single multiclass segmentation network or *n* uniclass segmentation networks, one for each class.

Summarizing the alternatives, they include the base loss formulas (crossE, IoU, dice), plus versions of dice and IoU that weight false positives and false negatives differently (exemplified here by IoU_xy_), dice or IoU with no background class (exemplified here as dice noBK), and finally the uniclass variation. Additionally, we can specify different combinations of α and β in the IoU_xy_ case. To limit the size of comparisons, we do not test alternatives dice_xy_ and IoU noBK.

### 2.3. Experimental Setup

The segmentation network architecture is a relevant factor for the quality of segmentation. For this work, we pick well-known segmentation networks, the U-NET [14], FCN [13] and DeepLabV3 [15]. The U-Net uses a 58-layer segmentation network with VGG-16 (7 stages, corresponding to 41 layers) for feature extraction (encoding). The FCN tested here also uses VGG-16 as encoder, and its total network size is smaller than UNet (51 layers). The decoder stages of U-Net are symmetric to the encoder stages, while FCN uses simple interpolation in the decoder stages. Both networks also include forward connections feeding feature maps from encoder to decoder stages. The two networks (U-Net and FCN) are the most-frequently used ones in segmentation of medical images. The third network, DeepLabV3, is a well-known segmentation network often used in object recognition applications that outperformed most competitors due to some innovations. It is the deepest network tested in this work, with 100 layers and uses Resnet-18 as feature extractor (8 stages, totaling 71 layers). DeepLabV3 incorporates two important segmentation quality enhancing improvements, the Atrous spatial pyramid pooling (ASPP) (improving segmentation of objects at multiple scales) and fully connected conditional random fields (CRF) for improved localization of object boundaries using mechanisms from probabilistic graphical models. All segmentation networks were pre-trained versions based on object recognition data.

The experiments reported in this work were preceded by a set of iterations tuning configurations to the best possible results. The final network training parameters after tuning, to be used in our experimental work, were: learning function stochastic gradient descent with momentum (SGDM), with an initial learning rate = 0.005, piecewise learning rate with drop period of 20 and learn rate drop factor of 0.9 (i.e., the learn rate would decrease to 90% every 20 epochs). Training iterations were 500 epochs; minibatch sz = 32; momentum = 0.9. The factor that most improved performance in our initial tuning prior to experiments was data augmentation, which we described before. A machine with a GPU NVIDIA G Force GTX1070 was used for the experiments.

Class balancing was applied in the pixel classification layer. Class balancing is a common operation in machine learning for datasets where classes have very different numbers of representatives (class imbalance). In medical images, class imbalance biases the result of the iterative backpropagation learning process to favor background over the target structures or organs. To illustrate the class imbalance problem, a classifier that classifies every pixel as background will guess correctly 95% of times if the background represents 95% of all pixels, yet it does not segment any structures or organs correctly. To solve this problem, class balancing multiplies the contribution of each pixel or class in the computation of the loss function by the inverse of its frequency in the whole dataset. Those class weights are added to the last layer, the pixel classification layer.

The experiments were divided into two phases. The first phase chose the best performing segmentation network among the three candidates, using the default cross entropy loss function. Using the best performing chosen network (DeepLabV3), we then tested the various loss functions. The loss functions used are cross entropy (crossE), IoU (IoU11) and IoUxy with different configurations of x and y, dice and dice without considering the background (dice noBK). In the case of IoUxy, we first test the following options: IoU11 = IoUxy with α = 1, β = 1, IoU1505 = IoUxy with α = 1.5, β = 0.5, IoU0515= IoUxy with α = 0.5, β = 1.5. Afterwards, we run a sensitivity experiment testing all combinations of α and β with steps 0.25. Finally, we compare multiclass versus n-uniclass segmentations. In what concerns metrics used to evaluate the quality of the resulting segmentations, we focused mostly our analysis on per-class IoU (JI), since it allows us to assess the quality of segmentation of each organ/lesion separately, and mean IoU over all classes.

## 3. Results

### 3.1. Choose Best-Performing Network

All experiments ran on independent test datasets after training and are the average over 5 cross-validation runs. For MRI and CT data, patient sequences were divided randomly into 5 folds such that each fold has 20% of all patients. This allowed us to run 5 experiments, each one considering one fold as containing the testing sequences and the remaining folds as training sequences (80% training/20% testing). Table 1 shows the IoU (JI) of UNet, FCN and DeeplabV3 (with cross-entropy crossE loss) for the MRI, CT and EFI datasets (Table 4 details example cross-validation runs for the MRI dataset).

Table 2 shows the results of different loss variations for MRI and EFI data, and Table 3 shows the corresponding results for CT data. The base loss functions tested were crossE, IoU and dice. Variants tested were no background (dice noBK) and different combinations of weights in IoU (different weights α and β applied to false negatives and false positives). Table 4 details cross-validation runs for the MRI dataset, to show that the difference of scores is statistically relevant. We report the mean IoU of each fold, average mean (IoU) over the 5 folds, the standard deviation, the 90% CI interval limits and the p-value (the p-value evaluates the null hypothesis that the differences observed between each of the loss functions and that of iou0515 might be purely by chance).

### 3.2. Loss Formula Weights: Sensitivity Run Using IoU_xy_ Loss Function

Since variations of weights were useful for MRI data, in this experiment we vary the alpha and beta coefficients of the denominator of IoU loss function ($\alpha FP_{i}+\beta FN_{i}$) using a step of 0.25 for the MRI dataset. Table 5 shows the evolution of the mean IoU for different values of alpha in one run.

### 3.3. Would It Be Worth Running by n-Uniclass Problems Instead of One Multiclass Problem?

Table 6 compares the scores of the multiclass problem with those obtained for n uniclass problems considering the MRI dataset (n = 4, one for each organ), and for the EFI dataset (n = 5, one for each lesion). The objective is to evaluate whether running n-uniclass segmentations, one for each organ/lesion, would improve or degrade scores. To compare the two options, the next experiment reports results of two runs: (1) multiclass segmentation (all organs/lesions in a single network); (2) uniclass segmentation for each organ/lesion separately. For the MRI dataset, multiclass scores were higher for any loss function: crossE, dice and IoU improved from (0.77, 0.73, 0.79) to (0.84, 0.86, 0.85) using multiclass. Looking at the details per organ, only the liver scores the same (crossE, dice) or better (IoU11) using the uniclass alternative. In the case of EFI, uniclass scores were also similar or lower than multiclass scores in most cases, and the average IoU scores are higher for the multiclass alternative as well.

## 4. Discussion

According to the results shown in Table 1, the best-performing network was DeepLabV3. Since UNet is a popular network for medical imaging, this result was surprising. As part of our future work, we are currently studying the details that contribute to this difference. The use of Resnet residue-based encoder network, ASPP and CRF should be important factors when compared with UNets’ VGG-16. In the same table, we can also see that MRI organ scores are much better than EFI lesions scores. In EFI, the background and optic disk score high (90% to 97%), but lesions score much lower (13 to 52% using crossE). Most of the background is fairly constant, and the optic disk also has relatively constant location and shape. In MRI, organs score reasonably high (77% to 86%). Organs in MRI are also located in similar places and have similar conformations, although their shape varies between different slices. Eye fundus lesions, on the other hand, are much smaller and/or have varying conformations and sometimes also lack adequate contrast. In general, there are also more errors near region borders in MRI and EFI, so that small/thin regions have higher error rates relative to their area. By modifying the loss function, Table 2 shows that scores of lesions in EFI improved from (13% to 52% using crossE) to (18% to 61% using dice noBK).

From Table 2 (comparison of scores of loss alternatives for MRI and EFI data), and Table 3 (same for CT data), we conclude that the best performing loss function differed slightly depending on the dataset, but there were some common patterns. Dice was the best for the liver CT dataset (91% versus 86% of crossE); IoU0515 (i.e., IoU with modified FP and FN weights) was the best performing loss variant for the MRI dataset (90% versus 84% of crossE); Finally, dice with no background was the top performing alternative for EFI data (59% versus 53% of crossE). Evaluating only the base loss functions (no variations) over the three datasets, dice scores best (85% MRI, 57% EFI, 91% CT), followed by IoU (86% MRI, 53% EFI and 89% CT) and finally crossE (84% MRI, 53% EFI, 86% CT). This means that dice loss improved scores by an average of one percentage point (pp), 4pp and 5pp for MRI, EFI and CT datasets, respectively, when comparing with crossE. Considering weight variations (different weights to false positives and negatives, zero weight on background = noBK) the score improvement is larger. Tuning α and β weights of false positives vs. false negatives improved scores further in MRI by 6 pp for a total of 12 pp, while assigning zero weight to class background improved scores in EFI dataset by 4 pp for a total of 6 pp. The top improvement per class considering the best variations were 11% for the right kidney in MRI, 15% for soft exudates in EFI and 9% for the liver in the CT dataset.

Table 4 and Table 5 show additional details. Table 4 details cross validation runs for the MRI dataset, showing that the differences observed between for instance IoU_xy_ and cross entropy are statistically significant. Table 5 shows how scores varied in MRI as α and β weights on false positives versus false negatives are modified in steps of 0.25. It shows that values of α between 0.5 and 0.75 scored highest for the MRI dataset.

The last experiment (Table 6) concerned evaluating whether replacing the multiclass problem by a set of n-uniclass problems, one for each non-background class, would improve or worsen the results, using the MRI dataset and the EFI dataset as well. Multiclass scores were better for any loss function: in the case of MRI, crossE, dice and IoU improved from (0.77, 0.73, 0.79) to (0.84, 0.86, 0.85) using multiclass. Looking at the details per organ, only the liver scores the same (crossE, dice) or better (IoU11) using the uniclass alternative. For the EFI dataset, taking the example of dice loss, we have multiclass (0.16, 0.28, 0.61, 0.51, 0.91) and uniclass (0.12, 0.25, 0.60, 0.36, 0.91). The conclusion is that the multiclass problem scored higher in general. The potential advantage of considering the uniclass problem would be that loss would only have to optimize for one class. However, organs/lesions that are not the target in each independent run can be more easily confounded with other organs/lesions that are now part of the background in that run (e.g., in the case of MRI, left and right kidney, or spleen with left kidney). The conclusion is that multiclass segmentation was better.

The final conclusions from the previous experiments are: (1) dice is the best in average over the three datasets when considering base loss functions without modifications, but IoU has scores similar. Cross entropy (crossE) was worse than both; and variations were useful in different ways in different datasets. While dice was the best in CT of the liver, IoU with specific weight modification was the best in the MRI dataset and dice without background was the best alternative in the EFI dataset. From those results, we conclude that dice should be used, but also that it is useful to consider different variations (dice_xy_ and dice noBK), tuning for a specific context. Another important conclusion from our experiments is that the single multiclass problem is preferable to expressing and solving *n* uniclass problems.

In what concerns generalization of our conclusions, we were careful to run multiple datasets and independent experiments, therefore, the results should be generalizable to other multiclass medical imaging problems in general.

## 5. Comparison with Related Work on MRI

For completion, in this section we review briefly results obtained by other authors segmenting MRI and CT scans (results for lesions segmentation on EFI images were reviewed briefly in related work section). Table 7 and Table 8 show the IoU reported by related MRI and CT segmentation approaches by other authors. Table 8 compares our scores with those of a few other approaches running on the same MRI dataset as ours (therefore, directly comparable), where we can see that our best performing approach was superior to those compared. Table 8 shows a broader picture of scores reported in other works, which implemented enhanced networks with architectural modifications added to improve segmentation quality of CT and MRI scans of abdominal organs. These works use different datasets from ours, and many of them segment CT instead of MRI, therefore they are not directly comparable to our results, however it is interesting to analyze their scores. In those results [39,40], achieved highest scores in segmentation of MRI images, and Hu et al. [41,42] obtained the best scores for CT. The results we obtained in this work, in spite of using only a general-purpose segmentation network and not testing other architectural modifications that were proposed in each of the works referenced in Table 8, are still “competitive”. Most importantly, they can be experimented with in future work with any of those works. Note also that, in general, in Table 8 segmentation of CT scans achieved better top scores than segmentation of MRI scans.

## 6. Conclusions and Future Work

The loss function is an important part of optimization in deep learning-based segmentation of medical images. It is important to analyze the effects of loss alternatives and whether they differ depending on datasets. In this paper, we investigate how the most popular loss functions (cross entropy, IoU and dice) and variations based on differently weighting factors compare in three different datasets. The objective was to find common patterns and to investigate if the variations that can be introduced in the base formulas can contribute to improve segmentation scores.

We have discussed metrics, loss functions and variations. Taking three different medical image segmentation problems we quantified the quality of loss, evaluating how the three popular loss functions compare in different settings, and how a set of variations affect the result. Experimentally, we firstly needed to choose the top-scoring network, considering UNet, FCN and DeepLabV3. We have concluded that DeeplabV3 outperformed the other two. Then we ran a set of experiments to explore how loss functions and their variations influence scores. Dice was the best in average over the three datasets, but we also concluded that variations were very useful in different ways in different datasets. In particular, we found that differently weighting of false positives and false negatives improved scores significantly for the MRI data, while removing class background from the loss formula improved scores significantly for the EFI dataset. However, these improvements were dependent on the dataset, hence we conclude that it is worth tuning the loss function taking into consideration these variations to adapt to the medical imaging context. We also analyzed how characteristics of different structures influence scores and how loss modifications can help overcome difficulties related to those characteristics. Finally, we compared single multiclass problem versus *n* uniclass problems in the MRI data.

There are a number of open challenges for future work that result from this work: one challenge is to determine why, based on architectural features, Deeplab3 outperforms UNet and FCN. This involves understanding the contribution of using a residue-based encoder (Resnet in DeepLabV3) versus VGG-16 (tested in UNet and FCN), as well as the contribution of other architectural features (e.g., ASPP and CRF of DeepLabV3). Another challenge is to understand what factors influence different scores of different variations in different medical imaging contexts. A direct extension of the work presented in this paper is to apply differentiated weights to other loss functions, and to extend the study to other advanced loss functions. However, the most important future challenge is how to improve quality of segmentation of the most difficult small and varying conformance targets, such as lesions in eye fundus images.

## Figures and Tables

**Table 1:** Intersect-over-the-union (IoU) of segmentation networks with base crossE loss (MRI, CT and EFI).

MRI Data	DeepLabV3	FCN	UNET	CT Data	DeepLabV3	FCN	UNET	EFI Data	DeepLabV3	FCN	UNET
Background	0.99	0.99	0.98					Background	0.97	0.89	0.75
Liver	0.86	0.86	0.74	Liver	0.86	0.77	0.75	Microaneurysms	0.13	0.02	0.01
Spleen	0.82	0.74	0.73					Hemorrhages	0.24	0.23	0.10
Rt Kidney	0.77	0.78	0.75					Hard Exudates	0.52	0.20	0.08
Lt Kidney	0.81	0.77	0.78					Soft Exudates	0.41	0.29	0.08
								Optic Disc	0.90	0.83	0.26
Avg IoU	0.85	0.83	0.80						0.53	0.31	0.21

**Table 2:** IoU of segmentation network DeepLabV3 with diff. loss functions, two datasets (MRI, EFI).

MRI	CrossE	IoU	IoU	Iou	Dice	Dice noBK	EFI	CrossE	IoU	Dice	Dice noBK
α β	-	1 1	1.5 0.5	0.5 1.5	-	-					
BackGround	0.99	0.99	0.99	1.00	0.99	0.99	Background	0.97	0.98	0.98	0.98
liver	0.86	0.84	0.69	0.88	0.87	0.84	Microaneurysm.	0.13	0.17	0.16	0.18
spleen	0.82	0.84	0.80	0.87	0.80	0.81	Hemorrhages	0.24	0.1	0.28	0.32
Rt kidney	0.77	0.82	0.77	0.88	0.81	0.82	Hard Exudates	0.52	0.61	0.61	0.61
Lt kidney	0.81	0.74	0.73	0.85	0.76	0.79	Soft Exudates	0.41	0.49	0.51	0.56
							Optic Disc	0.90	0.91	0.91	0.90
avg	0.84	0.86	0.82	0.90	0.85	0.85	avg	0.53	0.53	0.57	0.59

**Table 3:** IoU of segmentation network DeepLabV3 on CT data.

	CrossE	Iou11	Iou0515	Dice	Dice noBK
BackGround	0.98	0.96	0.98	0.99	0.98
liver	0.75	0.82	0.76	0.84	0.79
avg	0.86	0.89	0.87	0.91	0.89

**Table 4:** IoU of segmentation network DeepLabV3 for diff folds (CVi = cross validation fold) on MRI.

Mean (IoU)	CV1	CV2	CV3	CV4	CV5	Avg	stdev	Avg − CI	Avg + CI	*p*-Value
CrossE	0.843	0.834	0.829	0.833	0.836	0.835	0.006	0.831	0.842	0.000007
dice	0.848	0.851	0.864	0.849	0.852	0.853	0.007	0.845	0.859	0.00015
iou11	0.836	0.875	0.855	0.876	0.857	0.860	0.019	0.838	0.876	0.007
iou0515	0.879	0.901	0.903	0.895	0.881	0.892	0.011	0.871	0.892	-

**Table 5:** IoU of segmentation network DeepLabV3 for different values of alpha in MRI.

**Alpha**	0	0.25	0.5	0.75	1	1.25	1.5	1.75	2
**MeanIoU**	0.63	0.82	0.89	0.88	0.87	0.84	0.83	0.79	0.16

**Table 6:** IoU achieved with multiclass vs. uniclass on MRI and EFI.

MRI	Multiclass			Uniclass			EFI	Multiclass		Uniclass	
	CrossE	IoU	Dice	CrossE	IoU	Dice		CrossE	Dice	CrossE	Dice
BackGround	0.99	0.99	0.99	-	-	-	Background	0.97	0.98	-	-
liver	0.86	0.84	0.87	0.86	0.89	0.87	Microaneurysm.	0.13	0.16	0.16	0.12
spleen	0.82	0.84	0.80	0.58	0.62	0.52	Hemorrhages	0.24	0.28	0.31	0.25
Rt kidney	0.77	0.82	0.81	0.72	0.50	0.79	Hard Exudates	0.52	0.61	0.43	0.60
Lt kidney	0.81	0.74	0.76	0.70	0.67	0.79	Soft Exudates	0.41	0.51	0.45	0.36
							Optic Disc	0.9	0.91	0.87	0.91
avg	0.84	0.86	0.85	0.77	0.73	0.79	avg	0.53	0.57	0.45	0.45

**Table 7:** Comparing to IoU of related approaches (CHAOS dataset).

MRI JI = IoU	Liver	Spleen	R Kidney	L Kidney
teamPK [24]				
U-Net	0.73	0.76	0.79	0.83
V19UNet	0.76	0.79	0.84	0.85
V19pUNet	0.85	0.83	0.85	0.86
V19pUnet1-1	0.86	0.83	0.86	0.87
deeplabV3 iou 0.5/1.5	0.88	0.87	0.88	0.85

**Table 8:** IoU as reported in some related approaches (MRI and CT).

MRI JI = IoU	Liver	Spleen	R Kidney	L Kidney
[20]	0.84	0.87	0.64	0.57
[40]	0.90(LiverNet)	-	-	-
[39]	0.91	-	0.87	0.87
CT JI = IoU	Liver	Spleen	R Kidney	L Kidney
[43]	0.938	0.945		
[44]	0.85	-		
[19]	0.88	0.77		
[41]	0.92	0.89		
[42]	0.96	0.94	0.96	0.94
[45]	0.9	-	0.84	0.80
[23]				
F-net	0.86	0.79	0.79	0.80
BRIEF	0.74	0.60	0.60	0.60
U-Net	0.89	0.80	0.77	0.78
[21]	0.90	0.87	0.76	0.84

## Data Availability

Data is contained within the article itself, code used for coding loss is in https://github.com/pedronunofurtado/codingLOSS.
